# A Little Knowledge

**Published:** 1888-01-14

**Authors:** 


### The Doctor's Pulpit.

### "A LITTLE KNOWLEDGE."

"A little learning is a dangerous thing.
Drink deep, or taste not the Pierian spring."

Mr. Pope was of opinion that a little "learning" was dangerous; and many persons, misunderstanding the significance of the judgment, have misapplied the quotation, so that now we often hear of the exceeding danger, not of a little "learning," but of a little "knowledge." We will not attempt to draw any nice distinctions between "knowledge" and "learning," but it is perfectly well-known to any man who has any knowledge of Pope that what he meant when he used the word "learning" was not mere knowledge, and especially not knowledge of matters of fact, but academic culture—learning in the old-fashioned and still general acceptation of the term.

It is wonderful how wide is the influence of a proverbial expression (such as this has become) which sounds as if it contained a great deal of sense, and still more wonderful that the proverb should have such a long lease of authoritative life. It goes to prove how very few men and women there are in the world who possess any true originalty of mind. There is a sense, a perfectly obvious sense, in which Pope was right when he said: "A little learning is a dangerous thing." It is often dangerous because it makes men see things disproportionately and erroneously, and not seldom fills them with a foolish vanity and self-assertiveness which are exceedingly disagreeable. Men, in fact, of a little learning are sometimes great nuisances. But is it generally true, is it indeed *often* true, that a little knowledge of matters of fact is dangerous? Concerning this some will say that it is a matter of opinion. No! It is not a matter of opinion: it is a matter which can easily be brought to the test of experiment; and experiments, duly performed with sufficient frequency, are, we take it, generally convincing to reasonable minds. If, for example, a man were to say he could fly from Dover to Calais, everybody would be convinced that he spoke truth who should see him actually do so five hundred times in succession. Experiment duly performed is not only the Baconian method of arriving at truth, but in common things it has been everybody's method from the days of Adam until now.

Let us submit the misquoted and misunderstood dictum of Mr. Pope in its misquoted and misunderstood sense, to the test of experiment. And first let us do as the metaphysicians very properly do, and "define our terms." A "little knowledge is dangerous," say the misquoters. What do they mean by a "little knowledge"? Isaac Newton believed and affirmed that he possessed but a very little knowledge. "Every schoolboy," as Macaulay says, knows the classical story about the child playing on the seashore, and we need not repeat it. Newton, we say, felt in his heart, as everyone must who engages in the studies in which that rare philosopher spent his life, that his "knowledge" was as nothing compared with the number, magnitude, and variety of the objects to be known. Clearly, then, the word "little," as applied to knowledge, is a relative term, and if a man is to seek no knowledge at all because he can only acquire a little on any given subject, all study and inquiry must for ever cease from among men—*Quid est absurdum*, as the geometry books say.

But without spending our energies on such high subjects as astronomy let us come down to more elementary and every-day topics. Is it not of service to a man to know how to plant potatoes, even though he may not be able to cultivate a whole farm profitably? Is that housewife a dangerous woman who can set on a button merely because she has not acquired the art and mystery of making a coat? Will anyone say that a probationer nurse ought not to be allowed to give a man his physic because she is not able to diagnose his aortic aneurism? Or that a first year's student ought to discontinue the study of elementary anatomy because he cannot ligature the carotid artery?

These, we submit, are experimental tests of the correctness and value of the ridiculous affirmation, that "a little knowledge is a dangerous thing." As a matter of fact, a little knowledge is generally a most useful thing; sometimes it may even be a vital thing; and, whether or no, in most cases it is all that the cleverest amongst us can boast of.

As this is a semi-medical journal the subject may be very clearly brought home to our minds by reference to the every-day work of medical men. Do we never meet with cases concerning which our knowledge may most properly be described as "little." And are there no methods of treatment, no drugs in the "Pharmacopœia," of whose action we know very little? Do medical men know nothing of the blind groping of their professional ancestors in past ages; and of the exceeding difficulty of obtaining full and exact knowledge even with all the modern aids of physiological laboratories, post mortem rooms, and the most skilful and intelligent clinical clerks and directing physicians.

In opposition to those who nourish their intellectual life on worn-out phrases and conventional philosophies, we affirm that a little knowledge is generally a most useful thing; that everybody must possess a little before he can become master of a great deal; that in relation to *all* knowledge, the most encyclopædic man possesses but little, and that the passage as misquoted from Pope and generally understood is mere parrot-like chatter.

Knowledge is almost infinitely valuable, and for common purposes it cannot be too varied or extensive. "Knowledge is a defence," said Solomon. The expression is peculiar and full of significance. "A defence"—against what? Against all manner of evils. Against sharpers if you are in commerce; against climate, grubs, unsuitable manures, bad seeds and a thousand other dangers if you are in agriculture; against contagious fevers, drain throats, typhoid, typhus, diphtheria, and many other enemies more alarming than the midnight burglar, more terrible than the fiercest of wild beasts if you are a dweller in a great city.

The profound and accurate knowledge of the specialist may be left to the specialist. No man who does not devote his life to special labours can hope to thoroughly master such studies as astronomy, chemistry, biology, or medicine. But any intelligent man may find much that is interesting and practically useful in all of them. There are certain subjects on which none of us can afford to be entirely ignorant; and surely one of these is our own individual selves. Even as a matter of mental culture and scientific interest, a general knowledge of the structure and functions of the human organism is what an intelligent man should naturally desire. But such knowledge is capable of highly important practical applications. He who knows himself and his capabilities well has all the advantages over his ignorant competitor in life's battle that the experienced general has over the tyro. Instead of warning men off the field of knowledge by such feeble rattles as misquoted and misunderstood proverbial or poetical utterances, we would say, "Better is a little knowledge than none; happy is the man who has his mind stored with an unlimited variety."

